# When Limb Surgery Has Become the Only Life-Saving Therapy in FOP: A Case Report and Systematic Review of the Literature

**DOI:** 10.3389/fendo.2020.00570

**Published:** 2020-08-21

**Authors:** Esmée Botman, Sanne Treurniet, Wouter D. Lubbers, Lothar A. Schwarte, Patrick R. Schober, Louise Sabelis, Edgar J. G. Peters, Annelies van Schie, Ralph de Vries, Zvi Grunwald, Bernard J. Smilde, Jakko A. Nieuwenhuijzen, Marieke Visser, Dimitra Micha, Nathalie Bravenboer, J. Coen Netelenbos, Bernd P. Teunissen, Pim de Graaf, Pieter G. H. M. Raijmakers, Jan Maerten Smit, Elisabeth M. W. Eekhoff

**Affiliations:** ^1^Department of Internal Medicine Section Endocrinology, Amsterdam Bone Center, Amsterdam Movement Sciences, Amsterdam UMC, Vrije Universiteit Amsterdam, Amsterdam, Netherlands; ^2^Department of Anesthesiology, Amsterdam UMC, Vrije Universiteit Amsterdam, Amsterdam, Netherlands; ^3^Department of Rehabilitation Medicine, Amsterdam UMC, Vrije Universiteit Amsterdam, Amsterdam, Netherlands; ^4^Department of Internal Medicine Section of Infectious Diseases, Amsterdam Movement Sciences, Amsterdam UMC, Vrije Universiteit Amsterdam, Amsterdam, Netherlands; ^5^Department of Radiology and Nuclear Medicine, Amsterdam UMC, Vrije Universiteit Amsterdam, Amsterdam, Netherlands; ^6^Medical Library, Vrije Universiteit Amsterdam, Amsterdam, Netherlands; ^7^Department of Anesthesiology, Jefferson Health System, Thomas Jefferson University, Philadelphia, PA, United States; ^8^Department of Urology, Amsterdam UMC, Vrije Universiteit Amsterdam, Amsterdam, Netherlands; ^9^Department of Neurology, Amsterdam UMC, Vrije Universiteit Amsterdam, Amsterdam, Netherlands; ^10^Department of Clinical Genetics, Amsterdam Bone Center, Amsterdam Movement Sciences, Amsterdam UMC, Vrije Universiteit Amsterdam, Amsterdam, Netherlands; ^11^Department of Clinical Chemistry, Amsterdam Bone Center, Amsterdam Movement Sciences, Amsterdam UMC, Vrije Universiteit Amsterdam, Amsterdam, Netherlands; ^12^Department of Plastic, Reconstructive and Hand Surgery, Amsterdam Bone Center, Amsterdam UMC, Vrije Universiteit Amsterdam, Amsterdam, Netherlands

**Keywords:** fibrodysplasia ossificans progressiva (FOP), surgery, heterotopic ossification (HO), [^18^F]NaF PET/CT, ACVR1 gene mutation

## Abstract

Fibrodysplasia ossificans progressiva (FOP) is a rare disease in which heterotopic ossification (HO) is formed in muscles, tendons and ligaments. Traumatic events, including surgery, are discouraged as this is known to trigger a flare-up with risk of subsequent HO. Anesthetic management for patients with FOP is challenging. Cervical spine fusion, ankylosis of the temporomandibular joints, thoracic insufficiency syndrome, restrictive chest wall disease, and sensitivity to oral trauma complicate airway management and anesthesia and pose life-threatening risks. We report a patient with FOP suffering from life-threatening antibiotic resistant bacterial infected ulcers of the right lower leg and foot. The anesthetic, surgical and postoperative challenges and considerations are discussed. In addition, the literature on limb surgeries of FOP patients is systemically reviewed. The 44 year-old female patient was scheduled for a through-knee amputation. Airway and pulmonary evaluation elicited severe abnormalities, rendering standard general anesthesia a rather complication-prone approach in this patient. Thus, regional anesthesia, supplemented with intravenous analgosedation and N_2_O-inhalation were performed in this case. The surgery itself was securely planned to avoid any unnecessary tissue damage. Postoperatively the patient was closely monitored for FOP activity by ultrasound and [^18^F]PET/CT-scan. One year after surgery, a non-significant amount of HO had formed at the operated site. The systematic review revealed seventeen articles in which thirty-two limb surgeries in FOP patients were described. HO reoccurrence was described in 90% of the cases. Clinical improvement due to improved mobility of the operated joint was noted in 16% of the cases. It should be noted, though, that follow-up time was limited and no or inadequate imaging modalities were used to follow-up in the majority of these cases. To conclude, if medically urgent, limb surgery in FOP is possible even when general anesthesia is not preferred. The procedure should be well-planned, alternative techniques or procedures should be tested prior to surgery and special attention should be paid to the correct positioning of the patient. According to the literature recurrent HO should be expected after surgery of a limb, even though it was limited in the case described.

## Introduction

Fibrodysplasia Ossificans Progressiva (FOP) is an extremely rare disease with heterotopic ossification (HO) occurring in muscles, tendons and ligaments (1–3). HO usually leads to immobility of the affected joint, resulting in wheelchair-dependence at an early age (4). A flare-up often precedes the formation of this ectopic bone (1–4). A flare-up can occur spontaneously, but can also be triggered by a trauma (2, 4). Because trauma causes flare-ups and therefore aggravates the disease, patients are instructed to be careful (e.g., do not engage in contact sports), to refuse intramuscular injections and to prevent any kind of surgery (5). In some cases, though, surgery is inevitable when a medical condition is life-threatening. Surgical procedures can be difficult as extensive HO throughout the body has led to ankylosis of joints and has changed the patient's anatomy, making proper positioning of the patient difficult (4). Also, the anesthetic procedures are complex. The jaw of the patient is often ankylosed and pulmonary function can be severely restricted. As a result, standard anesthesia techniques can often not be applied to FOP patients (4, 6, 7). We report a patient with FOP who underwent a through-knee amputation due to a life-threatening antibiotics resistant infection. The surgical, anesthetic and postoperative considerations and challenges will be discussed. In addition, a systematic review on surgical procedures of the limbs and the course of the postoperative disease activity in FOP patients undergoing limb surgery is described.

## Case Report

**Figure 1:**
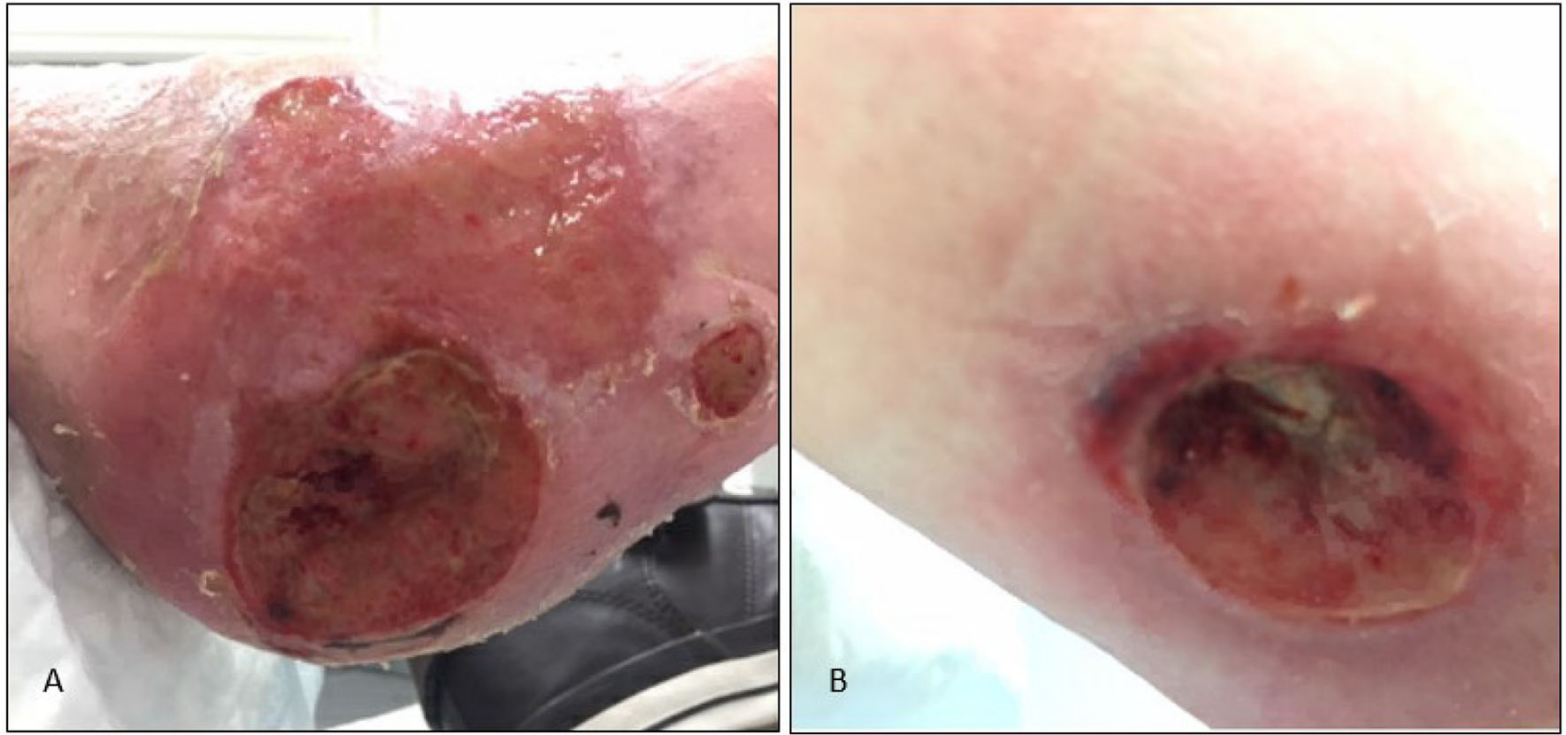
FOP patient with multiple incurable ulcers at the right lower extremity **(A)**. Ulcer located at the right calcaneus. Despite Intensive wound care, custom-made orthopedic shoes and targeted systemic and topical antimicrobial treatment, surgical intervention was unavoidable. Due to an ulcer on the calf **(B)** and proximal from the knee, a through-knee amputation was thought to be most favorable for adequate healing and to minimize tissue damage.

The patient, a 44 year old woman at the time of surgery, is known with the classical mutation (p.Arg206His) of FOP. Due to widespread HO throughout the body, she has been wheelchair bound and ADL (activities of daily life) dependent for over 25 years. Her joints are almost completely ankylosed except for ankles, toes, wrists and fingers i.e., cumulative analog joint involvement scale (CAJIS) score of 24 out of 30 (8). A recent pulmonary function tests showed a severely reduced Forced Expiratory volume in one second (FEV1) (0.6L, 25% of predicted) and forced vital capacity (FVC) (0.6L, 22% of predicted) with a normal Tiffeneau index (94%). This suggests a severely restrictive pulmonary function, compatible with marked chest wall rigidity (9). In 2016 the patient recovered without sequelae from a cerebrovascular accident (CVA), for which she is on chronic anticoagulation therapy (thrombocyte aggregation inhibitor). In addition, her fifth digit of the right foot was amputated in 2001 because of an incurable osteomyelitis. This procedure has previously been described (10). The patient has been treated in our center since 2016 for recurrent skin infections of the right lower leg and foot as a result of progressive chronic ulcers. A neuropathic pain syndrome, clinically confirmed by the neurologist, was thought to be the cause of allodynia in the right lower leg. Repeated pressure on the skin while sitting in her wheelchair contributed to formation of these ulcers. Initially, the ulcer at the foot led to recurrent skin and soft tissue infections of the right lower leg, with good response to antimicrobial treatment. Wound care led to improvement of the ulcer, but edema complicated healing. Intensive wound care, application of tailored wound dressing, and systemic treatment with antimicrobial agents with high bio-availability, targeted at cultured bacteria found in biopsies of the wound surface, resulted only in temporary improvements of wound healing. Custom-made shoes were manufactured to locally decrease pressure on the (pre)ulcer sites. With especially the combination of rigidity of the body and the wheelchair which can be adjusted into different positions turned out to be challenging in the use of these shoes. Initially it led to an improvement of the ulcers. But, unfortunately, after years of treatment the ulcers and infections progressed to chronic osteomyelitis with multidrug resistant microorganisms (including *Pseudomonas aeruginosa*) in visible and palpable bone in the wound surface (Figure 1). We expected her to develop a life-threatening sepsis in the near future. In a multidisciplinary FOP team, consisting of an endocrinologist, infectious disease specialist, pulmonologist, surgeon, anesthesiologists and rehabilitation specialist, the case was thoroughly discussed. The team concluded that amputation of the infected part of the lower leg was the only life-saving option. The patient was well-informed about the risks of the anesthesia, surgery and the risk of FOP activity after surgery and consented for a surgical procedure.

### Anesthetic Management

**Figure 2:**
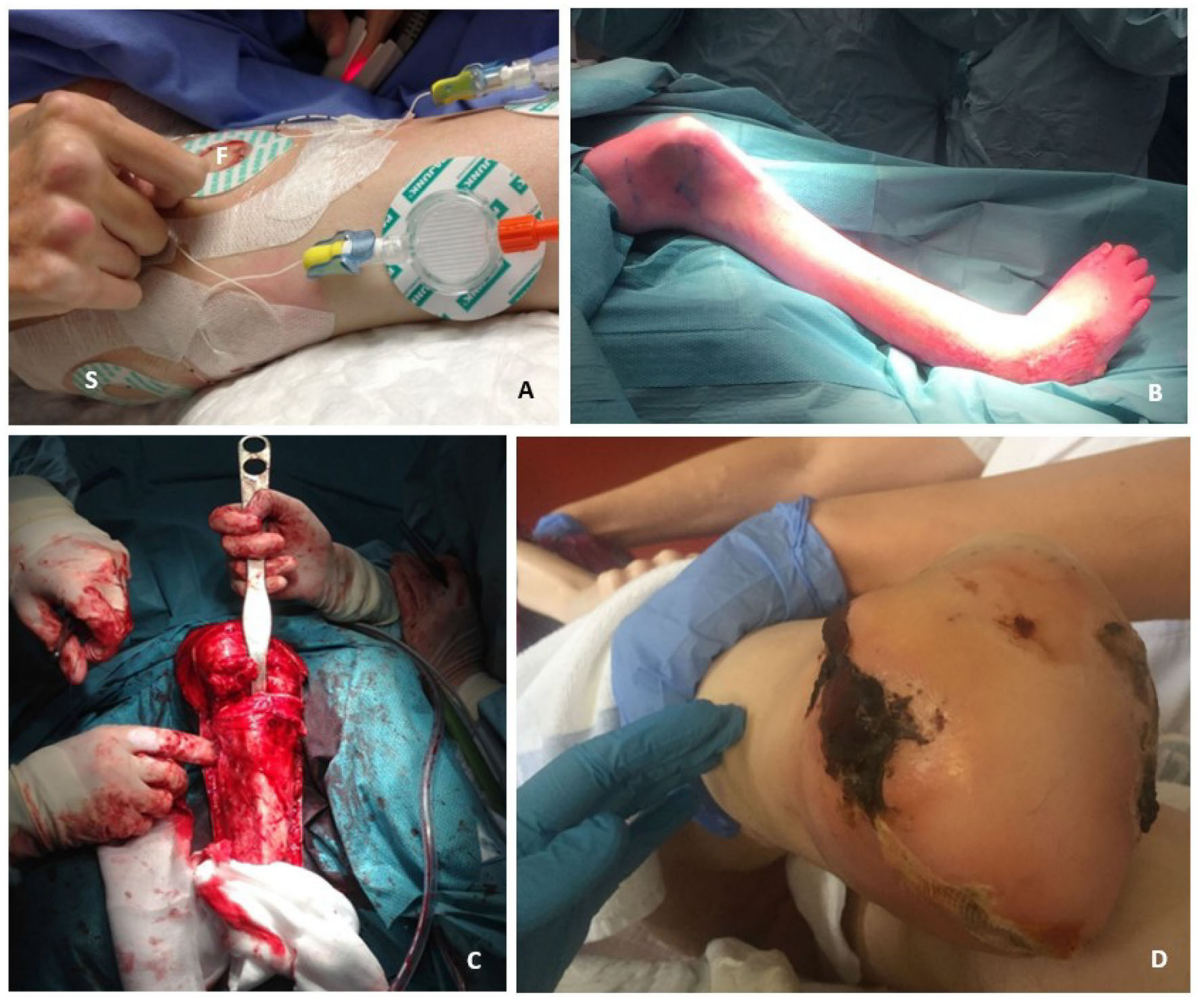
Anesthetic and surgical management of a through-knee amputation in an FOP patient. **(A)** Two nerve block catheters, i.e., the femoral (F) and the sciatic nerve block catheter (S), which were both already placed and tested at the preoperative day. The picture shows the antero-lateral aspect of the patient's right leg. The femoral nerve block catheter is positioned at the ventral aspect of the leg, whereas the sciatic nerve catheter is positioned at the lateral aspect of the leg. **(B)** The patient was carefully positioned on the theater table to prevent any tissue damage that might cause FOP disease activity. The positioning was challenging due to ankylosis in the hips and knees, resulting in the position shown in the picture. **(C)** Surgical procedure was performed carefully to minimize tissue damage that might cause a flare-up. **(D)** The skin flap and gastrocnemius muscle transposition were designed to opposite each other to prevent overlapping scars and minimize the chance of fistula formation due to expected wound healing issues. Lateral of the stump an area of necrosis developed, but healed with supportive care.

Anesthesiologists of our FOP expertise center in Amsterdam managed the anesthetic care. General anesthesia was intentionally avoided as airway management appeared rather challenging in this patient with severely impaired mouth opening (<2 mm). Moreover, mechanical ventilation was expected to temporarily cause a decline in pulmonary function, potentially leading to a ventilation-perfusion mismatch or barotrauma, and rendering weaning from mechanical ventilation impossible. Regional anesthesia was therefore selected as the preferred technique. Two peripheral nerve block catheters were placed at the femoral and sciatic nerve on the pre-operative day (Figure 2A). Damage to surrounding tissues was not completely avoidable, but kept to a minimum by using ultrasound guidance. The femoral nerve was easily identified in the femoral triangle. The identification of the sciatic nerve with ultrasound, however, was challenging because of an altered anatomy caused by HO (e.g., altered landmarks and aberrant course of the nerve). Eventually the sciatic nerve was identified and approached at the subgluteal level. To prevent inflammation at those sites, 40 mg methylprednisolone was administered over the two nerve block catheters. Pre-operatively, 12 ml ropivacaine 0.375% was injected in the catheters. The ropivacaine spread around the nerves as confirmed by sonography. Nerve block effectiveness was confirmed using cold discrimination tests prior to commencement of surgery. Surgery was initiated and anesthesia was judged adequate for the initial part of the procedure. The patient remained conscious and responsive throughout the procedure, but started to report some discomfort once surgery reached deeper tissue planes. The regional anesthesia was therefore supplemented by intravenous bolus titration of midazolam and s-ketamine, and inhalation of a mix of 50% N_2_O and 50% O_2_ via face mask. Herein, midazolam served as light anxiolytic and amnestic sedative, and to prevent psychomimetic side effects of s-ketamine. S-ketamine served as systemic analgesic without cardiovascular and respiratory depression. The N_2_O-inhalation induced additional analgesia, supplementing the analgesic effects of the regional anesthesia. Together, this ensured adequate analgesia and patient comfort for the remainder of the surgery, with a spontaneously breathing, responsive patient. Postoperatively, the patient did not recall having experienced any pain during the procedure. The nerve catheters were used postoperatively to administer continuous bupivacaine 0.125% for pain control, enabling to avoid the use of systemic opioids. The catheters were removed 8 days postoperatively when oral medication was sufficient to control pain.

### Surgical Management

Due to therapy-resistant infected ulcers 10 cm below the knee and more distally, it was decided to perform an amputation through the knee after an extensive discussion with our team and the patient. Thirty minutes prior to surgery, 30 mg of prednisolone was administered intravenously to prevent flare-ups. The surgery was performed by a surgeon affiliated to the FOP Expert Center of Amsterdam UMC. The positioning of the patient was challenging, due to complete immobility of the major joints (Figure 2B). Time was taken to carefully position the patient and soft pads were used to minimize pressure on the soft tissues. Once positioned, the patient was put in adjusted supine position and the knee joint was marked. A tourniquet was not used to avoid tissue compression that may induce a flare-up. As post-operative soft tissue healing complications were expected, the skin flap and gastrocnemius muscle transposition were designed to opposite each other in order to minimize the chance of deep infection and fistula formation. While most ligaments and the capsule of the knee joint were ossified, no abnormalities were observed in the knee joint itself (Figure 2C). As the patella was fused with the distal femur, it was left *in situ* to minimize tissue damage. Furthermore, the popliteal artery and nerve were difficult to identify initially, as the patient's leg was in a fixed position. This posed a potential risk in case of laceration of the vessel, but could be avoided by diligence. The gastrocnemius muscles were transposed forward and fixed near the patella region to cover the bone, to provide a vascularized bed and to protect underlying tissues in case of a future prosthesis (Figure 2D). The anterior skin of the proximal lower leg was fixed to the posterior skin at the level of the knee. Postoperatively, extra padding was applied between the lower extremities, to avoid pressure from the left knee on the wound of the stump. The patient developed partial skin necrosis laterally of the stump (Figure 2D), but healed with supportive care.

### Postoperative Management

**Figure 3:**
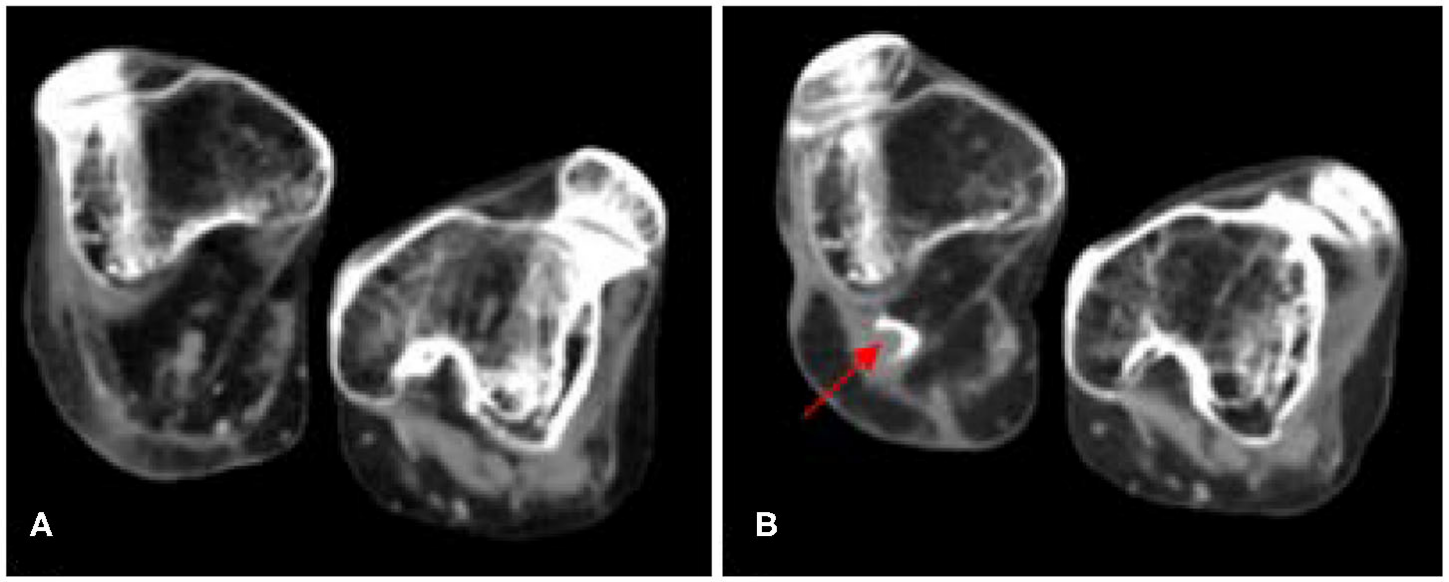
Axial Low dose CT-images at the level of the distal femur of a patient prior to and after a through-knee amputation of the right leg. **(A)** Eight months prior to the surgery. **(B)** Twelve months after the surgical procedure. Minor HO formed (4cc) on the right side posterior to the lateral femoral condyle (red arrow). FOP, fibrodysplasia ossificans progressiva; HO, heterotopic ossification; CT, computed tomography.

The patient's disease activity was closely monitored with ultrasound imaging and [^18^F]NaF PET/CT (sodium fluoride positron emission tomography and computed tomography). Ultrasound imaging was obtained daily to evaluate oedema at the surgical site and at the site of the anesthetic catheters. From day one until day fourteen, mild oedema was seen laterally from the stump by ultrasound. This oedema, however, did not progress over time. It was interpreted as a normal postoperative tissue reaction. To evaluate osteoblastic activity, an [^18^F]NaF PET/CT-scan was obtained 14 days after surgery, showing only a mild increased [^18^F]NaF-uptake (Standardized uptake value (SUV_max_): 6.4) at the base of the distal femur. Because the postoperative [^18^F]NaF uptake was only slightly elevated (11), it was decided not to administer extra prednisolone. A follow-up [^18^F]NaF PET/CT-scan was obtained 8 weeks after surgery, revealing minimal HO formation (4 cc) at the base of the femur (Figure 3). Another follow-up scan obtained 12 months after surgery, revealed no further progression of HO evaluated by CT. Interestingly, the patient's disease activity as evaluated by [^18^F]NaF-activity on PET, now showed an increased [^18^F]NaF-uptake at multiple sites of HO throughout the body, whereas in the previous 4 years there has not been any [^18^F]NaF activity nor a volumetric increase of HO as evaluated by CT. The quiescence of disease was in a period of progressive infectious ulcers and under continuous antibiotic therapy before surgery. After 14 days, the patient was transferred to a rehabilitation center. Since the patient was unable to see the stump and still feels the presence of her amputated lower leg, rehabilitation was needed to make her aware of the new situation and to find a new balance during transfers. The main goals of the rehabilitation were therefore to relearn the patient to make a standing transfer with help. The transfers were intensively practiced with the patient and her mother, who is an important informal care taker of the patient. Also, the electric wheelchair was adjusted to her new situation. After 4 months, the patient returned home. At the most recent follow-up, 14 months after the surgery, the patient was doing well. Now, the patient is under the care of the department of rehabilitation medicine at Amsterdam UMC exploring the possibilities of a cosmetic prosthesis of the lower limb.

## Systematic Review

Literature was systemically reviewed to identify cases in which FOP patients underwent surgery of a limb and the effect of the procedure on the disease activity. The literature search was performed based on the Preferred Reporting Items for Systematic Reviews and Meta-Analyses (PRISMA)-statement (www.prisma-statement.org).

**Figure 4:**
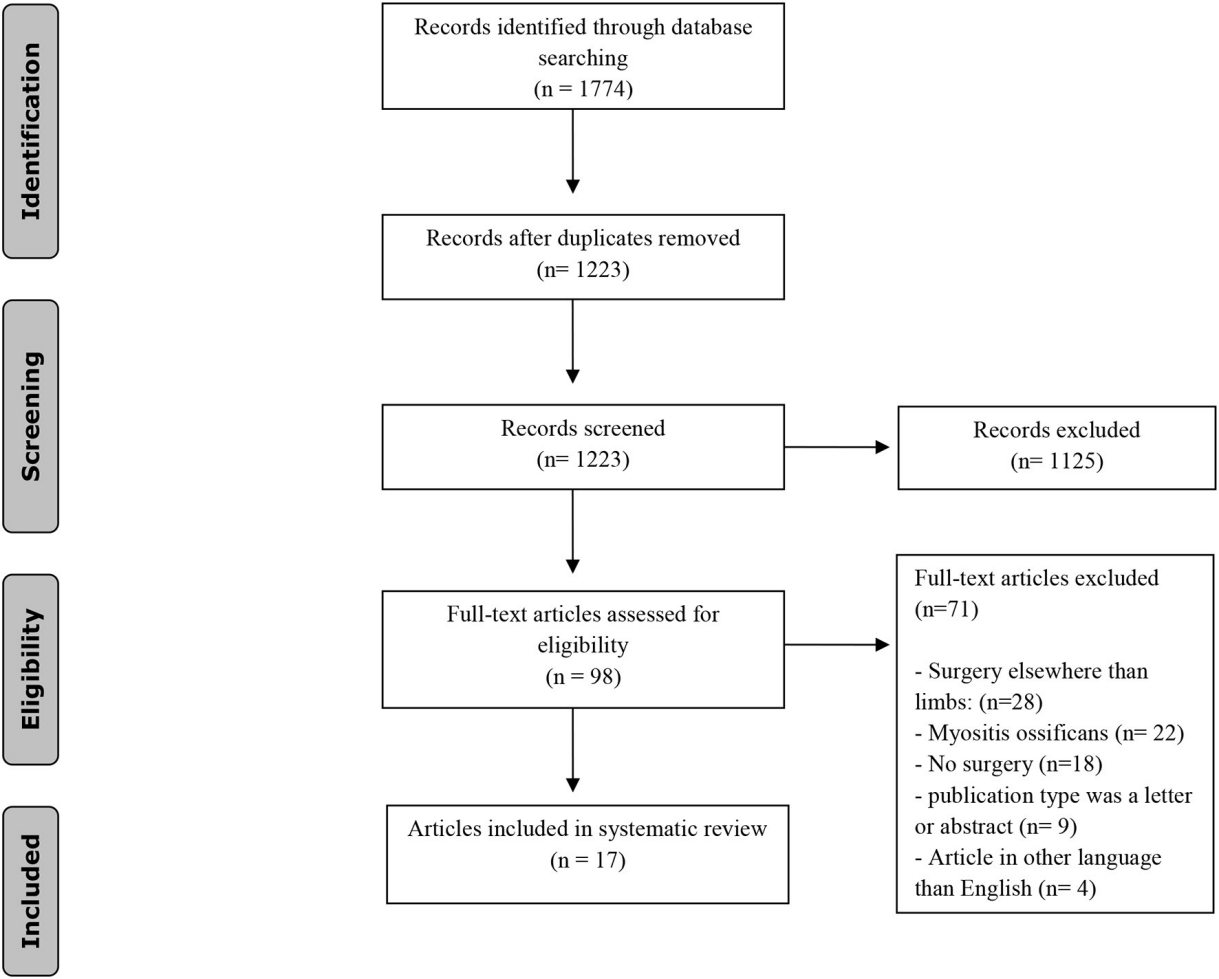
Flowchart of the study selection process.

To identify all relevant publications, we conducted systematic searches in the bibliographic databases PubMed and Embase from inception to May 2, 2019, in collaboration with a medical information specialist. The following terms were used (including synonyms and closely related words) as index terms or free-text words: “Myositis ossificans,” “Fibrodysplasia ossificans,” “Surgery,” “Anesthesia.” The references of the identified articles were searched for relevant publications. Duplicate articles were excluded. Only English articles were accepted. The full search strategies for all databases can be found in the Supplementary Material. Two reviewers (EB and ST) independently screened all potentially relevant titles and abstracts for eligibility. If necessary, the full text article was checked for the eligibility criteria. Differences in judgement were resolved through consensus. Studies were included when a surgical procedure of the limb and its outcome (either HO-formation or clinical outcome) were described. Patients of all ages were included, as well as all types of surgeries of the limb. The literature search generated a total of 1,774 references: 692 in PubMed and 1,082 in Embase. After removing duplicates of references that were selected from more than one database, 1,223 references remained. The flow chart of the search and selection process is presented in Figure 4. Seventeen articles described cases in which FOP patients underwent surgery for the upper and/or the lower limbs. In these seventeen articles, thirty two procedures were described in twenty patients. Ten procedures involved the upper limbs (12–19), twenty-two the lower limbs (Table 1) (12, 13, 19–28).

**Table 1:** Articles describing HO reoccurrence and/or clinical outcome after surgery of a limb in an FOP patient.

**References**	**Patient**	**Age (years)**	FOP- diagnosis (Y/N/U)*	**Body part**	**Reason surgery**	**HO (re)occurrence (Y/N/U)**	**(re)occurrence HO noted (months)**	**Duration follow-up (months)**	**Clinical improvement (Y/N/U)**	**Medication used pre-, peri- or postoperatively**
Benetos et al. (13)	1	14	Y	Shoulder	Unlock joint	Y	U	U	U	-
		18	Y	Hip	Unlock Joint	Y^1^	7	12	Y, improved mobility	Indomethacin 25 mg/3 dd, RT: 7 Gy in 1 fraction
Colmenares-Bonilla et al. (20)	2	11	Y	Knee	Unlock joint	Y^1^	1	60	N	Corticosteroids 30 mg/kg, Alendronate 10/mg/day
Connor et al. (14)	3	1	N	Shoulder	Remove swelling	Y	-	U	N	-
	4	6	U	Shoulder	Unlock joint	Y	U	U	N	-
Corfield et al. (15)	5	24	Y	Wrist	Improve position	Y^1^	3	3	Y: functional position	-
Duan et al. (21)	6	17	Y	Hip	HO induced claudication	U	U	24	Y: no claudication	-
Holmsen et al. (23)	7	20	Y	Hips	Unlock joint	Y^1^	2	24	N	EHDP 10 mg/kg/day
Jayasundara et al. (12)	8	47	Y	Shoulder	Unlock joint	Y^2^	U	U	Y: improved mobility	Bisphosphonates, indomethacin
				Hip	Unlock joint	Y^2^	U	U	N	Bisphosphonates, indomethacin
		52		Hip	HO induced pressure necrosis	U	-	U	N	RT: 26 Gy in 13 fractions
Kartal et al. (24)	9	13	N	Hip	Unlock joint	Y	1.5	12	N	-
		14	N	Hip	Unlock joint	Y	3	12	N	-
		15	N	Hip	Unlock joint	Y	U	12	N	-
Smith et al. (19)	10	34	U	Calf	Unlock joint	N^1^	-	U	U	EHDP 20 mg/kg/day
				Elbow	Unlock joint	U	-	U	Y: improved mobility	EHDP 20 mg/kg/day
	11	16	U	Foot	Unlock joint	Y	3	3	U	
		17		Foot	Unlock joint	Y	U	U	U	
		18		Hip	Unlock joint	Y	U	U	U	
		23		Foot	Unlock joint	Y^1^	36	36	U	EHDP 20 mg/kg/day
		U		Hip	Unlock joint	Y^1^	7	7	N	EHDP 20 mg/kg/day
	12	17	U	Hamstring	Unlock joint	Y	U	U	U	Prednisone 7.5 mg/day
		18		Biceps	Unlock joint	U	-	U	Y: improved mobility	EHDP 20 mg/kg/day
				Hip	Unlock joint	N^1^	-	24	Y: improved mobility	EHDP 20 mg/kg/day
	13	21	U	Hip^1^	Unlock joint	Y	2	5	N	EHDP 20 mg/kg/day
Kocyigit et al. (18)	14	15	N	Elbow	Unlock joint	Y	U	U	N	-
Matsuda et al. (28)	15	35	Y	Malleolus	Incurable ulcer	N	-	8	N	-
Nerubay et al. (25)	16	7	Y	Femur	Fracture	Y	U	12	N	-
Obamuyide et al. (16)	17	11	N	Axilla	Unlock joint	Y	U	U	N	-
Tiwari et al. (17)	18	2	N	Arm	Removal swelling	Y	U	U	N	-
Trigui et al. (26)	19	25	Y	Hip	Unlock joint	Y^1^	2	24	Y: functional position	Corticosteroids, bisphosphonates
Waller et al. (27)	20	23	Y	Hip	HO induced pain	U	-	U	Y: less discomfort	-

*HO reoccurrence is stated as YES when the article has specifically mentioned the reoccurrence of HO, YES^1^ when confirmed by X-rays and YES^2^ when confirmed by CT. FOP diagnosis is states as UNKNOWN when the confirmation of the diagnosis was not specifically mentioned in the article. FOP, fibrodysplasia ossificans progressiva; Y/N/U, Yes/no/unknown; HO, heterotopic ossification; RT, Radiotherapy; Gy, gray; EHDP, Ethylene Hydroxydiphosphonate*.

### Procedures on the Upper Extremities

All ten surgeries performed on the upper limbs were done to remove either an undiagnosed swelling or mature HO. The reoccurrence of HO was described for eight of the ten procedures (12–15, 18, 26) and the clinical outcome for nine cases (12, 14, 15, 18, 19, 26). Reoccurrence of HO was observed in all eight procedures, however, in four of the nine procedures for which the clinical outcome was described, a clinical improvement was noted (12, 15, 19). Clinical improvement in one patient was due to a better position of the joint (15), whereas three other cases describe an improved movement in the joint after surgery (12, 19). For these cases, though, the follow-up period was 5 to 6 months. The time for HO to redevelop after surgery was only described in only one case, i.e., 3 months (15). In the majority of the cases, the method to detect reoccurrence of HO and its extensiveness was not described. In all reports, neither the anesthetic management nor the selected operative method were discussed.

### Procedures on the Lower Extremities

Of the twenty-two described lower limb surgeries, performed in sixteen patients, twenty procedures were performed to remove HO and restore joint mobility (12, 13, 19–21, 23, 24, 26, 27). In one case, however, surgery was needed for fracture management (25), and in another case the operation was to close a chronic ulcer with skin grafts (28). In all but three cases in which HO reoccurrence was described (19/22), the removal of HO was complicated by reoccurrence of HO at the operated site (12, 13, 19, 20, 23–26, 28). Two of these three cases without any HO recurrence involved more than just the skin, however, adequate follow-up data on these cases are lacking (19). The time before HO was noticed ranged from 4 weeks to 36 months (13, 19, 20, 23, 24, 26). Despite reoccurrence, however, five of the sixteen cases in which clinical outcome was described, a clinical improvement after surgery was found (13, 19, 21, 26, 27). Two surgical procedures were done because of compression of HO on surrounding tissues, and resulted in less pain (21, 27). In two cases mobility was not restored, but a better position of the joint was achieved, increasing functionality (20, 26). In only one patient there was an actual improvement of mobility of the operated joint (13). The anesthetic management was mentioned, but not discussed in detail, in three case reports. Surgery to unlock the hip was performed under general anesthesia, whereas surgery on the knee joint was done under a subarachnoid block.

### Perioperative Medication to Prevent HO

Medication was used prior, during or after the procedure in 15 of the 32 surgeries (12, 13, 19, 20, 23, 26). In twelve of these cases bisphosphonates were used in attempt to halt (re)mineralization (12, 19, 20, 23, 26). Bisphosphonates were given as monotherapy (*n* = 8), or combined with non-steroidal anti-inflammatory drugs (*n* = 2) or corticosteroids (*n* = 2). The other treatments given were either NSAIDs combined with one fraction of radiotherapy (*n* = 1), subsequent fractions of radiotherapy (*n* = 1) or corticosteroids (*n* = 1) (12, 13, 19). For eleven of those fifteen procedures the effect of the procedure on HO reoccurrence was described: ten were followed by HO reoccurrence. The one case in which there was no reoccurrence, the duration of follow-up is unknown (19). Outcomes in the group without treatment (*n* = 17) were described for fifteen procedures: fourteen were followed by HO. The one case without reoccurrence was a superficial surgical procedure involving a skin graft for an ulcer on the malleolus (28).

## Discussion

Although any kind of surgery is highly discouraged in FOP patients due to an increased risk of flare-ups and progression of the disease, this case demonstrates that in a life-threatening situation–an operative procedure can be considered and managed successfully even in severely affected patients. It requires the assembly of a multidisciplinary FOP-dedicated team with knowledge of the disease and preparations made in anticipation of complications that may occur. In the current case, the timely and detailed preparation on the multidisciplinary team and the innovative techniques employed throughout the perioperative period assured the benign outcome of the surgical procedure. Because there is no effective treatment available to stop the formation and progression of HO, surgical procedures are highly discouraged as standard care of FOP (5, 29). Even small traumata–e.g., biopsies—can cause sufficient damage to the muscle and trigger a flare-up with subsequent HO formation (30). In the described case, surgery was the only life-saving option: it was judged that the patient was unlikely to survive the rapidly increasing, progressive infections of her leg due to antibiotic resistant organisms after many years of treatment. Surprisingly, a negligible amount of HO formed after the through-knee amputation, possibly due to a period of silent disease activity before and at the time of the operation. The reason for the quiescent disease in this patient is not known. One hypothesis is that, as it is known that the immune system plays a role in the pathogenesis of HO (31), the chronic inflammation and antibiotic use could have suppressed disease activity. Interestingly, 12 months after the surgical procedure disease activity was noted at various sites with HO. This could be the result of a normalized level of inflammation, or a systemic, late effect of the surgical procedure itself. Based on case reports in literature describing limb surgeries, where postoperatively HO formation was observed in almost 90% of the cases (12–21, 23–28), it was expected that clinically relevant HO would form. It should be noted, that over 90% of the published limb surgeries were performed to remove HO. Only in two patients (7%) HO did not reoccur after the removal of HO. Both patients received bisphosphonate treatment (19). Due to the absence of the effect of bisphosphonate treatment in nine others, it is more likely that the good result in those two can be attributed either to an incomplete follow-up time or due to limited imaging modalities as both cases are reported in 1976 (19). Removal of HO might be complex when it has formed within a muscle or when it has fused with normal skeletal bone. Removal of HO can therefore be considered as a high impact procedure which triggers HO formation. In our case a through-knee amputation was performed which is a procedure with relatively limited trauma to muscles because the procedure does not affect normal skeletal bone and it mainly involves the origin and insertion of muscles and tendons. In addition, when possible, ankylosed bone parts were left *in situ* to minimize tissue damage. To limit the extent of HO formation after surgery, it has been suggested to administer corticosteroids as a prophylaxis for four consecutive days after surgery (5). Objective data on the effectiveness of glucocorticoids in flare-ups are lacking. But based on empirical data, it is believed that it reduces oedema and may cause symptom relief (4). Glucocorticoids are currently the only treatment available for FOP. Corticosteroids, however, also interfere with wound healing. Therefore, in the current case, they were only administered pre-operatively. Hopefully, an effective treatment will be available to halt the formation of HO in the near future. To date, four potential drugs are tested in a clinical trial: Palovarotene, Garatosmab, Rapamycin and Saracatinib (32–35). Once found effective in preventing HO formation, surgical treatment might be an option to unlock joints or to safely operate an FOP patient for any other condition under an umbrella of one (or a combination) of these drugs. Besides the impact of the surgical procedure and the attempt to suppress FOP activity with glucocorticoids, the anesthetic management is another major concern and challenge in FOP patients. Regional anesthesia techniques (peripheral nerve blocks) involve punctures causing tissue trauma with increased risk of flare-ups, and these are therefore considered contraindicated. Likewise, neuraxial (spinal or epidural) anesthesia is not recommended for the following reasons. First of all, the spine is often involved in the disease and thus inapproachable for puncture. Secondly, the puncture itself might trigger HO formation, which could compress the spinal cord (5). Therefore, general anesthesia is generally recommended for FOP patients. General anesthesia requires airway management and frequently mechanical ventilation, both of which can be extremely challenging in FOP patients (36, 37). FOP patients often have jaw ankylosis, making conventional direct laryngoscopy or even video-laryngoscopy impossible for tracheal intubation. Moreover, even in the absence of a temporomandibular joint (TMJ) ankylosis, direct laryngoscopy is discouraged because hyperextension of the neck is limited–if not impossible–due to fused cervical vertebrae and in addition, overstretching of the TMJ joint or vertebral facet joints during tracheal intubation might induce a temporomandibular joint flare-up (5). Therefore, fiberoptic naso-tracheal intubation is preferred in all FOP scheduled for general anesthesia (5). This would have been possible in the current case, however, the risk of general anesthesia was deemed unacceptably high. The patient suffered from a severely restricted pulmonary function due to a completely immobile thoracic cage (7, 9). It was anticipated that high inspiratory airway pressures would be needed during mechanical ventilation to maintain adequate gas exchange. This can lead to over-distention of alveoli causing pulmonary barotrauma (38). Other challenges that were anticipated were a ventilation-perfusion mismatch and difficulties in weaning form mechanical ventilation. In addition, FOP patients are known to have impaired thoracic flexibility and weakened respiratory muscles predisposing to ineffective coughing, with an increased risk of mucus retention and infection (5). Therefore, a regional anesthesia approach was chosen, with ultrasound guidance to identify structures and to limit tissue trauma. Glucocorticoids were locally injected via the placed nerve block catheters in an attempt to prevent a flare-up. Since regional anesthesia alone was insufficient to ensure complete analgesia and patient's comfort, systemic drugs were added. As these drugs might induce apnea, it is important to monitor the patient closely and keep high-flow nasal oxygen standby in case support of oxygenation is needed (39, 40). To conclude, based on the literature it was almost certain that HO would form as a response to a surgical procedure of a limb. In the current case, HO was indeed formed, but even 12 months after surgery the volume of the formed HO minimal. It is hypothesized that the patient's silent disease activity and the continuous antibiotic treatments might have influenced this. If surgery needs to be performed, it is important that it is performed by a multidisciplinary team with knowledge about FOP and after carefully weighing the surgical benefits against the challenges and risks of both the anesthetic and surgical procedures for the FOP patient.

## Data Availability Statement

The datasets supporting the conclusions of this article will be made available by the authors, without undue reservation, to any qualified researcher.

## Ethics Statement

The authors have obtained informed consent from the patient to share data and images.

## Author Contributions

EB and EE: study design and data analysis. EB, ST, EE, JS, PS, WL, and LAS: study conduct. EB, EE, ST, and RV: data collection. EB, EE, JS, WL, and LAS: data interpretation. EB, EE, LAS, PS, WL, and JS: drafting manuscript. ST, LAS, PS, WL, EP, AS, RV, BS, JN, MV, DM, NB, JC, LS, BT, PG, PR, JS, and EE: revising manuscript content. EB, ST, LAS, PS, WL, EP, AS, RV, LS, BS, JN, MV, DM, NB, RV, JC, BT, PG, PR, JS, and EE: approving final version of manuscript. EE: takes responsibility for the integrity of the data analysis. All authors contributed to the article and approved the submitted version.

## Conflict of Interest

The authors declare that the research was conducted in the absence of any commercial or financial relationships that could be construed as a potential conflict of interest.
