# Supplementary material for: When Limb Surgery Has Become the Only Life-Saving Therapy in FOP: A Case Report and Systematic Review of the Literature
Source: Front Endocrinol (Lausanne). 2020 Aug 21;11:570. doi: 10.3389/fendo.2020.00570 (PMC7472799; doi:10.3389/fendo.2020.00570)
Supplement: Supplementary file 1 [file Table_1.DOCX]

**Supplementary material**

PubMed Session Results (2 May 2019)

| Search | Pubmed Query – May 2, 2019 | Items found |
| --- | --- | --- |
| [#3](https://www.ncbi.nlm.nih.gov/pubmed) | #1 AND #2 | 692 |
| [#2](https://www.ncbi.nlm.nih.gov/pubmed) | "Surgical Procedures, Operative"[Mesh] OR "Anesthesia"[Mesh] OR surger*[tiab] OR surgical*[tiab] OR operation*[tiab] OR operative*[tiab] OR perioperati*[tiab] OR anesthe*[tiab] OR anaesthe*[tiab] OR incis*[tiab] OR extract*[tiab] OR excis*[tiab] | 5,076,112 |
| [#1](https://www.ncbi.nlm.nih.gov/pubmed) | "Myositis Ossificans"[Mesh] OR myositis ossificans[tiab] OR ossifying myositis[tiab] OR fibrodysplasia ossificans[tiab] OR ossifying fibrodysplasia[tiab] OR FOP[tiab] OR Munchmeyer*[tiab] | 2,937 |

Embase Session Results (2 May 2019)

| Search | Embase Query – May 2, 2019 | Items found |
| --- | --- | --- |
| [#3](https://www.ncbi.nlm.nih.gov/pubmed) | #1 AND #2 | 1,082 |
| [#2](https://www.ncbi.nlm.nih.gov/pubmed) | 'surgery'/exp OR 'anesthesia'/exp OR surger*:ab,ti,kw OR surgical*:ab,ti,kw OR operation*:ab,ti,kw OR operative*:ab,ti,kw OR perioperati*:ab,ti,kw OR anesthe*:ab,ti,kw OR anaesthe*:ab,ti,kw OR incis*:ab,ti,kw OR extract*:ab,ti,kw OR excis*:ab,ti,kw | 6,866,560 |
| [#1](https://www.ncbi.nlm.nih.gov/pubmed) | 'ossifying myositis'/exp OR 'myositis ossificans traumatica'/exp OR 'fibrodysplasia ossificans progressiva'/exp OR 'myositis ossificans':ab,ti,kw OR 'ossifying myositis':ab,ti,kw OR 'fibrodysplasia ossificans':ab,ti,kw OR 'ossifying fibrodysplasia':ab,ti,kw OR FOP:ab,ti,kw OR Munchmeyer*:ab,ti,kw | 3,855 |
